# Big Data in Laboratory Medicine—FAIR Quality for AI?

**DOI:** 10.3390/diagnostics12081923

**Published:** 2022-08-09

**Authors:** Tobias Ueli Blatter, Harald Witte, Christos Theodoros Nakas, Alexander Benedikt Leichtle

**Affiliations:** 1 Department of Clinical Chemistry, Inselspital—University Hospital Bern, 3010 Bern, Switzerland; 2 Laboratory of Biometry, University of Thessaly, 384 46 Volos, Greece; 3 Center of Artificial Intelligence in Medicine (CAIM), University of Bern, 3010 Bern, Switzerland

**Keywords:** digitalization, clinical chemistry, artificial intelligence, interoperability, FAIRification

## Abstract

Laboratory medicine is a digital science. Every large hospital produces a wealth of data each day—from simple numerical results from, e.g., sodium measurements to highly complex output of “-omics” analyses, as well as quality control results and metadata. Processing, connecting, storing, and ordering extensive parts of these individual data requires Big Data techniques. Whereas novel technologies such as artificial intelligence and machine learning have exciting application for the augmentation of laboratory medicine, the Big Data concept remains fundamental for any sophisticated data analysis in large databases. To make laboratory medicine data optimally usable for clinical and research purposes, they need to be FAIR: findable, accessible, interoperable, and reusable. This can be achieved, for example, by automated recording, connection of devices, efficient ETL (Extract, Transform, Load) processes, careful data governance, and modern data security solutions. Enriched with clinical data, laboratory medicine data allow a gain in pathophysiological insights, can improve patient care, or can be used to develop reference intervals for diagnostic purposes. Nevertheless, Big Data in laboratory medicine do not come without challenges: the growing number of analyses and data derived from them is a demanding task to be taken care of. Laboratory medicine experts are and will be needed to drive this development, take an active role in the ongoing digitalization, and provide guidance for their clinical colleagues engaging with the laboratory data in research.

## 1. Introduction

Laboratory medicine has always been one of the medical disciplines with the highest degree of digitalization. Since its emergence, automation, electronic transmission of results, and electronic reporting have become increasingly prevalent [1]. In addition, medical laboratories maintain extensive databases, not only with test results, but also with results from quality controls. Furthermore, they are usually equipped with elaborate quality management systems. It is, therefore, not surprising that laboratory medicine represents a paradigm discipline for the digitalization of medicine. In contrast, the latest developments in the data science field, such as artificial intelligence (AI) and machine learning (ML), have not yet found their way into laboratory medicine across the board. Nevertheless, the time is now. Three key ingredients for augmenting laboratory medicine have become available to researchers on a wider scale: learning and training algorithms, necessary computational power to run said algorithms, and high-volume data [2]. These latest and future developments of AI and ML in laboratory medicine, however, do not constitute the main focus of this experience-based opinion article, since several recently published reviews can offer an excellent overview [1,2,3,4,5]. We will, instead, highlight the principles required for high-quality, clinical, “big” data. Without solid data as a foundation, even the most refined algorithms will fail to draw reliable conclusions: “ex falso sequitur quodlibet”, or, put more coarsely, “garbage in, garbage out”. The manifold requirements and pitfalls for Big Data analysis in laboratory medicine and fields of application shall be reviewed below.

## 2. Definitions Surrounding Big Data

Since the terminology “Big Data” has gained traction in the late 1990s, driven by the need to address the increasing data collection both in the private and public sector, there has never been a general agreement upon understanding of the term [6]. Initial depictions of Big Data were observing the phenomena and stating the emergence of a new discipline, without a cohesive clarification on what the Big Data term encompasses [7]. A more formal definition, proposed recently, suggests three main V’s (Volume, Velocity and Variety) as key dimensions of Big Data with the added requirement for specially designed technologies and analytics to translate data into value [8]. Subsequently, different scientific disciplines have attributed and highlighted other dimensionalities to Big Data (e.g., Value or Veracity [9,10]), while neglecting previously mentioned dimensionalities, making a holistic semantic definition rather difficult [11,12]. Ultimately, the strength of the “Big Data” conceptualization lays in its analytics, where Big Data is translated into clinical value.

**Figure 1:**
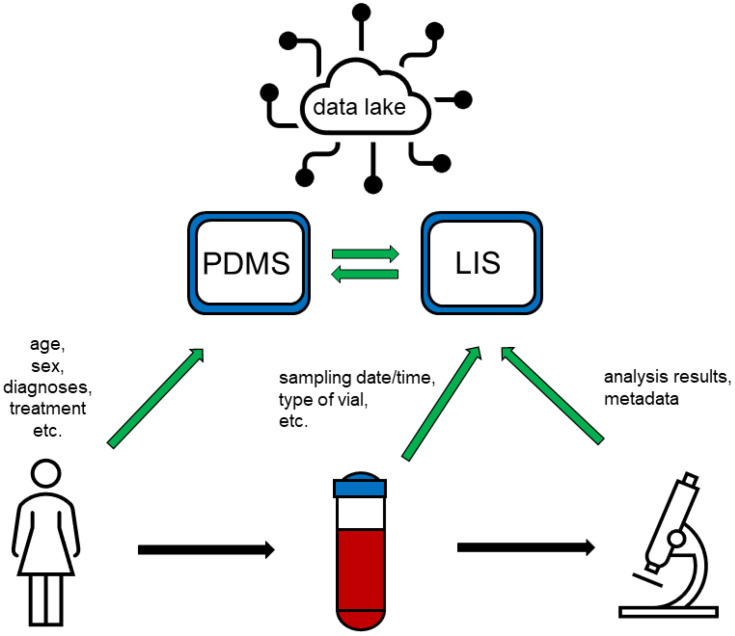
Patients’ data is entered into the patient data management system (PDMS), predominantly manually, while information about samples collected as well as about analyses conducted is entered into the laboratory information system (LIS), either manually or automatically. PDMS and LIS are connected and exchange parts of their stored data. Both systems feed a “data lake” comprising various types of data, which can be provided to researchers for Big Data applications.

Big Data analysis is the assessment of large amounts of information from multiple electronic sources in unison, by sophisticated analytic tools, to reveal otherwise unrecognized patterns [13]. Sources for Big Data are manifold, including data from laboratory information systems (LIS), “-omics” data from applications such as NGS (Next Generation Sequencing) or proteomics, and diagnostic data (Figure 1). If we consider “standard” laboratory analyses, e.g., clinical chemistry, haematology, or haemostasis testing, the lion´s share of analysis results consists of numerical results, possibly enriched with reference ranges. Notably, in terms of size—not comprehensibility from the point of view of a human observer though—all laboratory results of a medium-sized university laboratory fit on a standard hard disk. The situation is different with “-omics” data, which, depending on the technology, can comprise of several hundred megabytes to several gigabytes, be it NGS, proteome, or metabolome data [14]. A distinction must also be made between the usually very extensive, often proprietary raw data, and pre-processed data, which are often available in tabular form and correspond to standard multiplex laboratory analyses in terms of volume. Other fields with extensive data volumes are diagnostic diagrams, with information content that may be limited, but which require large storage capacities, when saved in the form of graphs; and diagnostic image data, e.g., from microscopy or MRI (magnetic resonance imaging). Another Big Data resource not to be underestimated is non-patient-related data, i.e., calibration and quality control data, which are often stored and administrated in specialized databases.

Laboratory data are best suited to the Big Data concept if they are enriched with clinical data from the hospital’s various IT systems.

## 3. Transforming Laboratory Medicine into Big Data Science

### 3.1. Requirements

Even though laboratory medicine databases constitute a rich source of data, frequently these are ill-suited for the application of data science techniques. Created to fit regulatory requirements instead of research purposes, most databases store data inefficiently and only for the minimally required retention period. Providing insufficient data quality for most research questions, databases are transformed into mere data dumps. So, what are the prerequisites for optimally usable laboratory medical data [15]? Central attributes data needs to have to be optimally suited for research use are summarized by the key word “**FAIR**”: Finable, Accessible, Interoperable, Reusable [16]. (cf. Table 1).

**Findable data** must be stored in a way that enables easy retrieval. For “standard” examinations, this is usually realised though a patient identifier (PID) and date, so individual results can be assigned to the respective patients and collection times. Depending on the organization of the laboratory, this is easier said than done. Potential pitfalls are, for example, that the same PIDs might be assigned to different patients in different branch laboratories, or that analyses conducted for unidentified emergency patients cannot be attributed to the correct person when their identity has been clarified. Additionally, results of different patients might be combined under a “collective” PID for research purposes. Moreover, data can be confusing when samples are registered with the planned collection date instead of the actual collection data, resulting in analysis time points prior to collection. Equipment for special examinations poses particular challenges to findability, as they are frequently not connected to the LIS. Here, the patient ID may be entered manually into the evaluation files in a way that does not conform to the standard, which can lead to confusion and incomplete entries. An example of this are “-omics” analyses: analytical devices routinely produce and output files too large for transfer and storage in the central LIS. Therefore, they need to be linked, preferably in a searchable manner to enable offline findability. Likewise, findability has to be addressed in the sharing of machine-actionable (meta)data online. Good metadata makes data findable. In web 1.0/2.0 approaches, this was addressed by the Linked Data Principles, a set of best practices when publishing structured data to the web [17]. These principles were however proposed before the emergence of FAIR, meaning that little emphasis was put on standardization and a variety of inherently different schemas were proposed [18]. One of the most recent efforts for making semantic artefacts, FAIR has been launched by the FAIRsFAIR project, where the authors list recommendations for findable (meta)data, highlighting the need for GUPRIs (Globally Unique, Persistent and Resolvable Identifiers), highly enriched and searchable (meta)data descriptions and, especially relevant for clinical laboratory sciences, the need to publish data and metadata separately [19]. Findability remains one of the most important aspects of the FAIRification of Big Data analysis, as a lack of appropriate metadata standards affects the availability of research data in the long term. A recent study observed decreased findability of UK health datasets over time [20], a trend also observed in a greater context of data-driven science, both in terms of the findability of datasets and the reachability of the responsible authors [21].

The **accessibility** of laboratory data can also be a challenge. LISs usually do not have freely accessible query functionalities because of regulatory requirements. Therefore, LISs that are not connected to central clinical data warehouses must be accessed through the laboratory IT personnel. This often leads to an enormous amount of additional work, since laboratory data are highly attractive for a variety of research projects [22]. For use in clinical data warehouses, the LISs must be electronically connected, and the data prepared via ETL processes (Extract, Transform, Load). This requires the use of universal web standards including HTTP (Hypertext Transfer Protocol), standardized data exchange formats (e.g., FHIR [23] and the semantic-based Resource Description Framework (RDF) [24,25]) and tools which allow querying respective data (e.g., SPARQL [26]). Additionally, data models like OMOP [27,28] or i2b2 [29] are in common use. In true FAIR fashion, LISs must present standard API (Application Programming Interface) with secure access protocols (e.g., SSL) for data management and retrieval [19]. Generally, the entire content of the databases is not transferred, but a limited subset of data (e.g., data records that can be clearly assigned to patients) is identified and transmitted. A special challenge in this context is posed by legacy systems that are solely operated in read-only mode, where the effort for the technical connection must be weighed against the benefit of the further use of the data contained. Moreover, as the available data for researchers grows, there need to be mechanism in place to enable privacy protection with the use of de-identification or anonymization algorithms. While textbook methods, for instance k-anonymity [30] or l-diversity [31], are often cited, they do not come without their limitations [32,33,34]. In this context, the question arises as to who is allowed to access the laboratory data and under what conditions. For example, data relating to infection serologies or staff medical service is particularly sensitive and requires careful data governance [35]. Another important aspect is the question of patient consent for research-project-access needs, to be restricted according to regulatory requirements [36]. The use of patient data in research in Switzerland is governed by the Federal Act on Data Protection (FADP 1992, art. 3c) and the Human Research Act (HRA RS 810.30). Notably, the governance of Big Data is not different from “regular” research data: A request on the disposal and use of sensitive data must be submitted to a cantonal REC (Research Ethics Committee). Big Data research proposes novel ethical concerns [37], mostly surrounding the notions of privacy (hindrance of individual reidentification) and consent (possibility to later revoke consent), where traditional ethics oversight practice is often unaware of the direct societal impact of their decisions [38]. A recent study in Switzerland showed that members of the seven Swiss RECs had broadly differing views regarding the opportunities and challenges of Big Data, citing insufficient expertise in big data analytics or computer science, to adequately judge the use of Big Data in clinical research [39]. This situation can become especially cumbersome for researchers when data from different institutions are merged—in this case, modern systems that work with secure multiparty computing and homomorphic encryption, such as the MedCo system, can be a promising approach [40]. Wirth et al. offer a great overview regarding privacy-preserving data-sharing infrastructures for medical research [41].

The next big and perhaps most important aspect for Big Data in laboratory medicine is the necessary **semantic interoperability**. This means that the individual data items must be clearly assigned semantically, ideally by means of standardized coding, e.g., along the lines of LOINC (Logical Observation Identifiers Names and Code). This represents an enormous challenge, which has been addressed in Switzerland, for example, by the L4CHLAB project [42]. It is not enough to identify laboratory analyses only by their trivial name (e.g., “potassium”)—the necessary granularity is defined by the requirements of the research projects based on it. Thus, a creatinine measurement of any kind may be sufficient as a “safety lab measurement” but be completely insufficient for a method comparison study or the establishment of reference intervals. It should be noted that currently there is no universal standard, as even LOINC does not specify, e.g., device manufacturer and kit version, which need to be coded additionally. Unique identifiers for medical devices, e.g., from the GUDID [43] or EUDAMED database [44], or type identifiers, e.g., from medical device nomenclatures such as GMDN [45] or EMDN [46], may enrich the LOINC system and increase its acceptance. Extensive preparatory work to address this issue has been done by the Swiss Personalized Health Network (SPHN), which established corresponding “concepts” [47]. Particular difficulties arise from historically grown LISs, which are often not structured according to the 1:1 principle of LOINC nomenclature, preventing a clean assignment of laboratory analyses to unambiguous codes. This must be considered especially when replacing and updating LISs, so that the master data remains future-proof and interoperable [14]. The use of advanced data models such as RDF is beneficial here, as it allows a data scheme to evolve over time without the need to change the original data [25]. In the university environment, the latest test technology might be employed, using analyses that do not yet have a LOINC code assigned, making it necessary to deviate accordingly. For the consolidation of large amounts of data from different sources, a high semantic granularity, which is necessary for individual questions, can be problematic, as equivalent analyses must be defined as such in order to enable comprehensive evaluations. Here, Minimum Information Checklists (MICs), stating the minimum requirements for quality and quantity to make data descriptions accurate and useful, could offer a needed standardization to track data quality from various sources [48,49]. It is essential that a core vocabulary features support for descriptions to be machine-readable RDF [50], closely linking the commonly used semantics in laboratory medicine with machine-actionable descriptions. The use of semantic web technologies, such as RDF, in the laboratory environment could also help to establish the common use of Electronic Lab Notebooks (ELNs) [51]. Notably, the application of suitable data formats facilitates, but by itself does not guarantee, actual interoperability of data sets from different data providers. Seemingly trivial details including spelling, cardinalities, datatypes, consistent use of GUPRIs, or measurement units must be carefully assessed. In the context of RDF, the Shapes Constraint Language (SHACL) allows the testing and validating of data against a set of predefined requirements [52]. These conditions (SHACL rules) constitute a “shape graph” against which the actual data (as “data graph”) is matched. The expression of complex constraints is facilitated by SHACL-extensions supporting SPARQL and JavaScript [53,54]. Despite the rise of user-friendly validation tools, semantic standards alone are not a “silver bullet” against data mayhem. In fact, even with maximum semantic care, the competence of experts in laboratory medicine remains in high demand. Different automated approaches for resolving the semantic heterogeneity when mapping different ontologies have been launched but still require human oversight [55,56]. For many researchers who come from non-analytical subjects, the differences in the meaning of the analysis codes are not obvious at first glance. Considerable misinterpretations can occur, e.g., calculation of eGFR from urine creatinine. Here, the laboratory holds responsibility since it has the necessary competence to avoid such errors.

The **reusability** of laboratory medical data depends to a large extent on the existence and level of detail of the associated metadata. This includes—as already mentioned—not only analysis-related data (mapped in the dimensions of LOINC) but also batch numbers, quality management data, and, if applicable, SPRECs (Sample PREanalytical Codes) [57]. In essence, everything that is or could be of importance for optimal replicability of the measurement results. It can be problematic that the metadata are stored in separate databases and cannot be provided automatically via the ETL processes, so that they can neither be exported nor viewed. Not only the (meta)data needs to be reusable but also the algorithms and data-processing scripts. With “FAIRly big”, a functional framework for retracing and verifying the computational processing of large-scale data based on machine-actionable provenance records, high performance could be observed regarding data sharing, transparency, and scalability, despite ignoring explicit metadata standards [58]. Reusability can also refer to the efficient use of statistical models that may arise using machine learning methodology. The latter may involve a feedback process, where the model is validated and even further calibrated as information arrives through the expansion of the database with fresh data. Potential pitfalls impairing reusability may include legislative limitations imposed by national research acts or legal ambiguities in Data Transfer and Use Agreements (DTUA) of multicentre cohort studies involving several data providers.

### 3.2. Risks

The use of laboratory medical data for Big Data analytics does not only have advantages but is also associated with a considerable number of risks: as all health data, laboratory values are worthy of special protection. As with all information compiled in large databases, there is an imminent risk of data leaks, especially if the data are accessible from the outside. Structured laboratory data can also be copied easily and quickly due to their small file size, so there is a considerable risk of unauthorized data duplication. Similarly, data governance must be ensured, which requires a comprehensive authorization framework—this is easier to implement in closed LISs. Another essential aspect is data integrity, which must be ensured in particular through the ETL process pipelines and also for further processing. LISs, as medical products, usually fulfil the necessary standards, but with self-written transformation scripts this may be different, so enforce a meticulous quality control. However, this has the advantage that non-data transfer-related errors can also be detected and deleted. In any case, certification of the IT processes is both sensible and costly. Post-analytics can also cause difficulties—the IT systems of the receivers (clinicians or researchers) must be able to handle the data formats supplied and must not alter or falsify their presentation. Another enormously problematic aspect is change tracking. In the LISs, laboratory tests are often identified by means of their internal analysis numbers—if changes occur here, e.g., due to the inclusion of new analyses, changes must be reported to the peripheral systems—preferably automatically and with confirmation of knowledge—otherwise serious analysis mix-ups can occur. Finally, when individual laboratory data are queried, the framework of the findings is no longer guaranteed—the analyses lose their context and, thus, their interpretability.

### 3.3. Chances

The introduction of “Big Data” technologies holds great potential for laboratory medicine, and some aspects will be specifically addressed here.

Setting up ETL processes inevitably leads to the detection of inadequacies in the structure and content of the laboratory’s master data. Frequently, LISs have grown over years and—although continuously maintained—are not organized in a fundamentally consistent manner. Before one can begin with the extraction and processing of laboratory data, the data organization, structure, and meta information must already be disclosed in the source system. A thorough review of this data is recommended to be carried out in the mother database, because tidying up is in any case necessary, which is quite obviously better done in the source system than in subordinate databases. Another important aspect is the necessary introduction of clear semantics—this is a laborious process that initially represents a large workload but is subsequently relatively easy to maintain. Many laboratories are reluctant to take on this effort—here, the diagnostics manufacturers are asked to supply the necessary codes (e.g., extended LOINC codes, see above) for the analyses they offer, e.g., in tabular form, which makes bulk import considerably easier and a matter of a few days. For researchers, in particular, it is also extremely helpful to have a data catalogue created in this context. Laboratory catalogues are often available electronically but are usually organized around request profiles, rather than individual analyses that are often of importance for research questions. The IT teams of the data warehouses will also be very grateful for appropriate documentation. This also offers the opportunity to make extensive metadata accessible and usable for interested researchers. Together with the introduction of semantics and data catalogues, transparent change tracking should be integrated, so queries in the data warehouses can be adapted accordingly, if, for example, analyses have changed, or new kits have been used. Change tracking is also clearly to be advocated from a good laboratory practice (GLP) point of view.

Another aspect of outstanding importance for laboratory medicine as a scientific subject is the visibility and documentability of the contribution of laboratory medicine to research projects. In the vast majority of clinical studies, laboratory data play an extremely important role, be it as outcome variables, as safety values, as quality and compliance indicators, or as covariates. With a transparent database and query structure, the use and publication impact of laboratory data can be shown more clearly and the position of the laboratory in the university environment as an essential collaboration and research partner can be strengthened. Other aspects include the improved use of patient data for research purposes—turning laboratory databases from graveyards of findings into fertile ground for research, an aspect that is certainly in the interest of patients in the context of improvement of treatment options. The improved indexability of laboratory data in large “data lakes” would also allow to link them to clinical data. Conversely, this also opens up completely new research possibilities for laboratory scientists, as the laboratory values no longer stand alone, but can be analysed in a clinical context. Last but not least, a cleanly curated database is an essential foundation for AI applications. It is like in most data science projects: 80% of the effort is data tidying, and 20% is the “fun part” of the analysis. Here the laboratories have to point out their very important, but little prestigious and extremely tedious role. They are essential partners in the vast majority of research collaborations.

### 3.4. Fields of Application

Big Data, with its technological environment, does not yet represent a translation into medical fields of application, but it should be regarded as a basis and facilitator for a large number of potential uses. Mainly applications come into consideration that already require a large amount of information to be processed and, thus, bring the human part of the evaluation pipeline to a processing limit. These include, of course, data-intensive “-omics” technologies, including not only pattern recognition in specialized metabolic diagnostics and new-born screening but also technical and medical validation and quality management. Further applications can be population-based evaluations such as the creation of reference value intervals. In the following, some of the potential fields of application are described.

An obvious field for Big Data technologies in laboratory medicine are “-omics” applications [59,60,61]. These have been developed for nucleic acid-based techniques as, e.g., genomics [62,63], transcriptomics [64], and epigenomics [65], as well as for mass spectrometry-based methodologies such as proteomics [66,67], metabolomics [68,69], lipidomics [70], and others. The particular challenges in this field include connecting the analysis systems to the corresponding data lakes—it is no longer possible to work with traditional database technologies and new approaches, for example, hadoop [71] become necessary. Even more than in the case of highly standardized routine procedures in classical laboratory medicine, metadata play an outstanding role in evaluability, comparability, and replicability. In addition, the raw data generated with these procedures are often formatted in a proprietary manner and are also of enormous size—comparable only with the data sets of the imaging disciplines. For retrieval, indexing and linking to the respective patient must be ensured; this can be achieved, for example, by linking tables of processed results instead of raw data output. The extent to which transformation and evaluation steps already make sense in the ETL process depends on the respective question, but following the FAIR principles, open file formats should be made available in addition to raw data, even if the transformation process is often accompanied by a loss of information (e.g., in mass spectrometry).

Moreover, in other diagnostic fields where a large number of different analyses have to be medically validated synoptically, Big Data technologies offer a good basis for the development of pattern recognition and AI algorithms, which not only help to automate workflows efficiently but also can recognize conspicuous patterns without fatigue and, thus, lead to a reduced false negative rate. New-born screening is a prime example of this [72], but complex metabolic diagnostics will also benefit from data that is machine learning ready—there is still considerable potential for development [73]. For algorithms to be registered as “medical devices”, the hurdles to be taken are fairly high, including proper assessment of potential risks, detailed software design specifications, traceability, data security, etc., just to name a few obligations to be compliant with the new “Medical Device Regulation” (MDR) of the European Union [74]. Moreover, to be used in hospital settings, data collection requires strict quality-management systems certified in accordance with ISO 13485 [75]. Currently, European notified bodies or other authorities such as the U.S. Food and Drug Administration (FDA) or the UK Medicines and Healthcare products Regulatory Agency (MHRA) have started to adapt guidelines for Good Machine Learning Practice (GMLP) for the development of AI and ML applications as medical devices or have overhauled their existing regulations [76,77,78]. We are now witnessing the clearance of the first AI-based algorithms for prediction and diagnostics for use with patients. The “IDx-DR” algorithm, which detects diabetic retinopathy from retinal images, is an inspiring example [79]. It was the first medical device using artificial intelligence to be approved by the FDA, in April 2018, and for use on the European market, in April 2019. [80,81] Data from a multi-centre study with 900 patients enrolled at 10 different sites were a cornerstone for the approval of the “IDx-DR” algorithm—a masterpiece, unthinkable without proper “Big Data” management [79].

Besides laboratory diagnostics itself, there are a large number of other fields of application for Big Data in laboratory medicine. For example, the field of quality management. Mark Cervinski notes that “modelling of Big Data allowed us to develop protocols to rapidly detect analytical shifts”—additionally, administrative and process-oriented aspects, such as optimizing turnaround time (TAT), can also benefit from Big Data [13]. Especially, since under a big workload, the main factor affecting TATs is not the verification step of test results but rather the efficiency of the laboratory equipment [82]. With the help of predictive modelling, TATs could be highlighted that are likely to exceed their allocated time. Furthermore, these highlighted TATs could potentially be relayed to the ordering clinician, allowing new levels of laboratory-reporting transparency.

Clinical-decision support systems are more oriented towards clinical needs and are essentially based on laboratory data. This can be in the context of integrated devices [83] or more- or less-complex algorithms that enable the integration of multimodal information and allow clinicians to quickly and reliably make statements about the diagnostic value of the constellations of findings. An example of this is the prediction of the growth of bacteria in urine culture based on urine-flow cytometric data [84].

Perhaps the most exciting field of application for Big Data in laboratory medicine, however, is predictive and preemptive diagnostics. With the help of laboratory data, probabilities for a variety of patient-related events can be calculated and, in the best case, therapeutic countermeasures can be initiated, so that the events do not occur in the first place. This can range from the prediction of in-house mortality, in the sense of an alarm triage [85,86], to the prediction of derailments in the blood glucose levels of diabetic patients [87]—the possible applications are almost unlimited.

## 4. Conclusions and Outlook

Laboratory medicine has always been a data-driven discipline—more so than ever with the advent of multi-parametric and “-omics” technologies. On the other hand, the discipline has been largely fossilized by a way of working that has remained almost unchanged for decades and by the specific requirements of clinicians and regulatory bodies for reporting findings [88]. This is especially true for routine clinical diagnostics, so opening up to “Big Data” represents a challenge that should not be underestimated. Yet, this openness represents the basis for modern technologies, in particular deep learning or artificial intelligence, which can bring diverse advantages not only for diagnostics but also for laboratory medicine as an academic and research-based medical discipline. Many steps that are required in the transformation of laboratory medicine data into “Big Data” [22] can be used for research make sense anyway for lean, efficient, sustainable, and complete data management and can lead to a cleansing and “aggiornamento” (modernization) of laboratory data. If laboratory medicine shies away from these developments, it will be degraded to a pure number generator in the foreseeable future or disappear completely as an academic subject in integrated diagnostic devices. On the other hand, the importance of comprehensive, quality-assured laboratory medical data and metadata for clinical research can hardly be underestimated. It is important to set standards for the openness, willingness to collaborate, and FAIRification of medical data. After all, health data is the new blood [89]—which can also revitalize laboratory medicine not only in a figurative sense.

## Figures and Tables

**Table 1:** Recommendations for the FAIRification (FAIR+) of laboratory data.

Requirements	Implementation
Findability	- Assign PIDs meaningfully. Each PID should uniquely identify a single patient, which needs to be consistent between branch laboratories with parallel systems. Develop solutions for unknown emergency patients, which allow correct assignment of test results when personal data is identified later on. Develop solutions for analyses conducted for research purposes. Avoid cumulative PIDs. - Record actual sampling time instead of planned sampling time. - Connect all analytical devices to the lab IT system to avoid manual entries. - Connect the lab IT system to the hospital’s central IT system to enable searches by clinicians and researchers.
Accessibility	- Protect lab data adequately with: secure data storage solutions. careful data governance. - Design ETL processes efficiently. - Consider the general consent status of patients and allow access to data accordingly. - Employ modern technical solutions such as multiparty computing and homomorphic encryption for merging data from different sites.
Interoperability	- Code analyses in a standardized manner, e.g., with LOINC codes. - Additionally, code the device manufacturer and kit version in a standardized way. - Code newly developed analyses in a homogenous way, even if no standardized codes are available yet. - Enable consolidation of data from different labs.
Reusability	- Provide detailed metadata to maximize reproducibility, including: LOINC codes. batch numbers. quality management data. SPREC codes.
+	- Offer your laboratory medicine expertise to clinicians and researchers, as no one knows the intricacies of your laboratory data better than you.

Abbreviations: ETL: extract—transform—load; lab: laboratory; LOINC: Logical Observation Identifiers Names and Code; PID: patient identifier; SPREC: Standard Preanalytical Code. + signifies the additional human resource (laboratory expertise).

## Data Availability

Not applicable.
